# Brachydactyly E: isolated or as a feature of a syndrome

**DOI:** 10.1186/1750-1172-8-141

**Published:** 2013-09-12

**Authors:** Arrate Pereda, Intza Garin, Maria Garcia-Barcina, Blanca Gener, Elena Beristain, Ane Miren Ibañez, Guiomar Perez de Nanclares

**Affiliations:** 1 Molecular (Epi)Genetics Laboratory, Hospital Universitario Araba-Txagorritxu, BioAraba, Vitoria-Gasteiz 01009, Spain; 2 Department of Genetics, Hospital Universitario Basurto, Bilbao 48013, Spain; 3 Department of Genetics, BioCruces Health Research Institute, Hospital Universitario Cruces, Barakaldo 48903, Spain; 4 Department of Radiology, Hospital Universitario Basurto, Bilbao 48013, Spain

## Abstract

Brachydactyly (BD) refers to the shortening of the hands, feet or both. There are different types of BD; among them, type E (BDE) is a rare type that can present as an isolated feature or as part of more complex syndromes, such as: pseudohypopthyroidism (PHP), hypertension with BD or Bilginturan BD (HTNB), BD with mental retardation (BDMR) or BDE with short stature, PTHLH type. Each syndrome has characteristic patterns of skeletal involvement. However, brachydactyly is not a constant feature and shows a high degree of phenotypic variability. In addition, there are other syndromes that can be misdiagnosed as brachydactyly type E, some of which will also be discussed. The objective of this review is to describe some of the syndromes in which BDE is present, focusing on clinical, biochemical and genetic characteristics as features of differential diagnoses, with the aim of establishing an algorithm for their differential diagnosis. As in our experience many of these patients are recruited at Endocrinology and/or Pediatric Endocrinology Services due to their short stature, we have focused the algorithm in those steps that could mainly help these professionals.

## 

Brachydactyly (BD) refers to a family of limb malformations characterized by shortening of the hands, feet or both [1]. It was added to the international Nosology and Classification of Genetic Skeletal Disorders in 2001, in the group of genetically determined dysostoses [2,3]. Different types of brachydactyly can be distinguished based on anatomic grounds, the most commonly used classification being that provided by Bell [4] and modified by Temtamy & McKusick [5]. Most types are rare, except for A3 (BDA3, OMIM#112700) and D (BDD, OMIM#113200) that have a prevalence of around 2% [1]. In this review, we focus on brachydactyly type E (BDE, OMIM#113300), which is rare and can be diagnosed as an isolated finding or as part of several genetic syndromes [1,5,6].

BDE encompasses variable shortening of the metacarpals/metatarsals, frequently with involvement of the phalanges [5]. Hertzog [7] classified BDE into three distinct varieties: type E1, with shortening of metacarpal IV, sometimes associated with shortening of metatarsal IV (possible involvement of an isolated metatarsal); type E2, with shortening of metacarpals IV and V (and metatarsals) associated with shortening of the distal phalanx of the thumb; and type E3, with various combinations of short metacarpals without phalangeal involvement [8]. However, these patterns are not held to exactly in all syndromes; as well as the distal phalanx of the thumb, others phalanges are often shortened.

In this review, we describe some common syndromes in which BDE is present, outlining their main clinical, biochemical and genetic characteristics (Additional file 1: Table S1), with the aim of establishing an algorithm for the accurate diagnosis of BDE in association with other features. Even if acrodysostosis and tricho-rinho-phalangeal syndrome cannot be considered as conditions with a pure brachydactyly E, as they have peculiar radiological features, sometimes they are misdiagnosed as syndromic BDE, so we will try to define the specific characteristics that distinguish them from all the other conditions.

One of these specific features is multihormonal resistance, mainly to pthyroid hormone (PTH) and thyroid hormone (TSH). We use this feature to classify the syndromes into two groups depending on the presence of multihormonal resistance as many of these patients are recruited at Endocrinology and/or Pediatric Endocrinology Services due to their short stature that leads to the analysis of hormonal profile.

## Isolated brachydactyly type E: *HOXD13* type (OMIM#113300)

While BDE is commonly reported as part of a syndrome, it occasionally appears as an isolated entity [9-11]. Four heterozygous mutations in the homeobox D13 gene (*HOXD13,* 2q31.1), two missenses and two nonsenses, have been identified as causative of isolated BDE [9-12]. In two of these four cases, an overlap between BDE and BDD (shortening of the distal phalanx of the thumb) was noted in the patients [9]. Other hand malformations that have been observed in combination with BDE are syndactyly of digits III and IV, synpolydactyly of finger IV, and long distal phalanges [9]. In addition, 21 independent mutations have been identified in *HOXD13*, most of them producing synpolydactyly (SPD; OMIM#186000), or in rare cases syndactyly, and other unspecified limb malformations [11].

**Figure 1:**
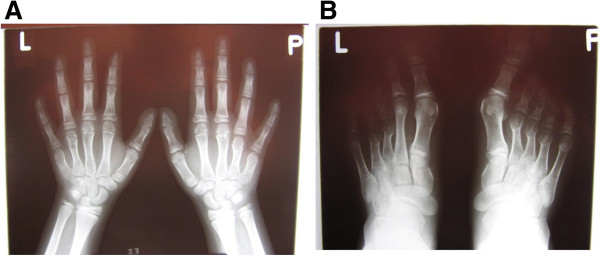
**Radiographs of hands (A) and feet (B) of a non adult individual with heterozygous mutation in *HOXD13*(OMIM#113300) (courtesy of Dr. Aleksander Jamsheer).** Shortening and widening of IV and V metacarpals are more evident in right; I, IV and V distal brachyphalangy, and mild clynodactyly of the distal phalanx of II and V, in left hand. In the feet, note asymmetrical shortening of the metatarsals: in right feet II, III, IV are shortened and in the left one, only III and IV. Note also, the clynodactyly of right hallux.

Regarding BDE caused by mutations in *HOXD13*, the pattern profile showed many variations between affected patients: most of them had shortening of metacarpals III and sometimes also IV and V (Figure 1A); and in the feet, shortening of the metatarsals IV was frequently seen, sometimes in combination with that of metatarsals I, III or V and broadening of the hallux (Figure 1B). In addition, little-finger distal phalanx hypoplasia/aplasia, lateral phalangeal duplication and/or clinodactyly of finger IV, and syndactyly of fingers III/IV were frequently observed [9,11,13]. Finally, in general, affected individuals had normal stature and no appreciable psychomotor developmental delay (Additional file 1: Table S2) [9,11,13,14].

## Brachydactyly type E as part of syndromes

### BDE with multihormonal resistance

#### Pseudohypopthyroidism

Pseudohypopthyroidism (PHP) refers to several distinct, but related, disorders in which resistance to pthyroid hormone (PTH) is the most prominent feature [15]. However, resistance to thyroid-stimulating hormone (TSH) and several other pituitary hormones that mediate their action through G-protein-coupled receptors is often present as well. As a result of PTH resistance, patients with PHP develop hypocalcaemia and hyperphosphataemia. Resistance seems to occur only in the proximal renal tubule, while the action of PTH is unimpaired in other target tissues, such as bone and the renal thick ascending limb [16-18].

**Figure 2:**
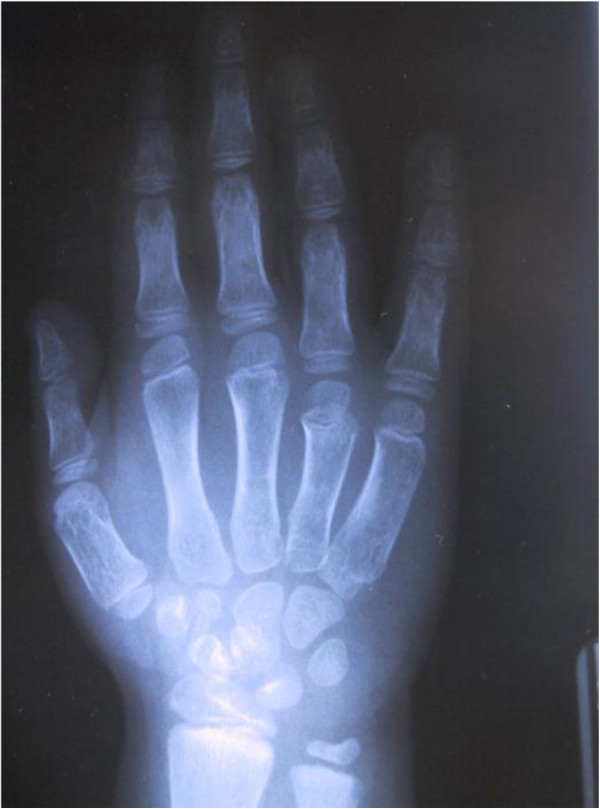
**Hand of a patient with pseudohypopthyroidism type Ia (OMIM#103580).** Note the shortening of first metacarpal and proximal phalanx of the thumb and more severe shortening of metacarpals IV and V (courtesy of Dr. Beatriz Garcia-Cuartero).

BDE is present in 70-78% of individuals with PHP [19]. There are, however, great differences in hand shortening: some patients display severe shortening of all hand bones, whereas commonly in others metacarpals and distal phalanges are more impaired than other segments. In particular, Poznanski *et al.* reported that the distal phalanx of the thumb and metacarpal IV were the most affected in 75% and 65% of patients respectively [20]. On the other hand, while de Sanctis *et al.* did not observe differences in prevalence of shortening, they reported that metacarpal V and distal phalanges I and IV were the most severely affected (85.7% < −2 SDS) (Figure 2) [19]. They also noted variations in the patterns of bone shortening between subjects with the same mutation, within the same family as well as among unrelated individuals [19]. Most publications on PHP only mention shortening of metacarpals III, IV and V or metatarsals, but it should be clarified that the shortening of these bones is usually more evident than shortening of phalanges; the latter may also occur, but is more difficult to assess because of the wide range of variation within the normal population [21].

PHP is associated with alterations in the *GNAS* locus, which maps to the telomeric end of the long arm of chromosome 20 (20q13.2-20q13.3) [22]. *GNAS* exemplifies a locus of high complexity [23]. One of its products is the α-subunit of the stimulatory heterotrimeric G protein (Gsα), a ubiquitous signalling protein that is essential for numerous different cellular responses. Gsα expression is biallelic in most tissues, but paternal Gsα expression is silenced in a few types of tissue (renal tubule, thyroid gland, hypophysis and gonads) [24-26] and this is important in the development of phenotypes associated with *GNAS* mutations. Maternal imprinting (and thus paternal expression) occurs on XLαs, the large form of Gsα, and two non-coding RNA molecules, the A/B transcript and the NESPas antisense transcript. On the other hand, paternal imprinting is seen on NESP55, a chromogranin like neuroendocrine secretory protein [27].

Heterozygous inactivating mutations within Gsα-encoding *GNAS* exons, leading to diminished activity, are found in patients with PHP-Ia (OMIM#103580). These patients have not only resistance to hormones but also the Albright’s hereditary osteodystrophy (AHO) phenotype (as an example, see Figure one at Miao et al. [28]) (Additional file 1: Table S3) [27]. This phenotype includes BDE (Figure 2), small stature, obesity with a rounded face, subcutaneous calcifications, and mental retardation [29]. Patients with AHO features but no evidence of hormone resistance are said to have pseudopseudohypopthyroidism (PPHP, OMIM#612463), and also carry heterozygous inactivating Gsα mutations. Maternal inheritance of such a mutation leads to PHP-Ia, AHO and hormone resistance, while paternal inheritance of the same mutation causes PPHP [27].

On the other hand, PHP-Ib (OMIM#603233) is another form of PHP which can follow an autosomal dominant (AD) trait (AD-PHP-Ib) or occur as a sporadic disorder. These patients present with PTH and sometimes TSH resistance, but resistance for other hormones is not found. Apart from this, patients often lack AHO features and exhibit normal Gsα activity in erythrocytes and fibroblasts. Nevertheless, there have been some reports of patients with mild AHO [30-33] and these individuals have diminished Gsa activity [34]. Generally, AD-PHP-Ib is caused by specific deletions in the syntaxin 16 gene (*STX16*) (*STX16del4-6* or *STX16del2-4*); which are associated with maternal inheritance and cause loss of methylation at *GNAS* exon A/B or 1A. Other form of AD-PHP-Ib, due to maternally inherited microdeletions of *NESP55* identified within *GNAS*, produces a loss of all maternal *GNAS* methylation imprints [35,36].

Sporadic PHP-Ib cases also display *GNAS* imprinting abnormalities that involve NESPas, XLαs and A/B. However, the genetic lesion, if any, underlying these epigenetic defects remains to be discovered and most of these cases could represent true stochastic errors in early embryonic maintenance of methylation [37-39]. Some of these patients have been shown to be affected by paternal uniparental isodisomy (pat20iUPD) involving part or the whole long arm of chromosome 20, which includes the *GNAS* locus [39-42]. A mild BDE and AHO phenotype has been reported in some studies [30,31,43]; in most cases, only metacarpal IV and/or V shortening was seen, although mild shortening of all metacarpals and some distal phalanges has also been described [33]. Sanchez *et al.* found BDE in 60% of patients with PHP-Ib in their study (no subjects presenting shortening of distal phalanx I). In two patients, BDE was combined with Madelung-like deformities (involvement of the distal ulna and radius) [43]. On the other hand, Mantovani *et al.* did not find any correlation between the severity of the AHO phenotype and methylation defects, their patients’ phenotype ranging from mild to severe AHO [33].

#### Acrodysostosis with multihormonal resistance (ACRDYS1, OMIM#101800)

pthyroid hormone-related protein and G-proteins are part of the same signalling pathway affecting cartilage differentiation and growth in the metacarpals. This could explain the similarity of PTHLH-related BDE, pseudohypopthyroidism and acrodysostosis.

**Figure 3:**
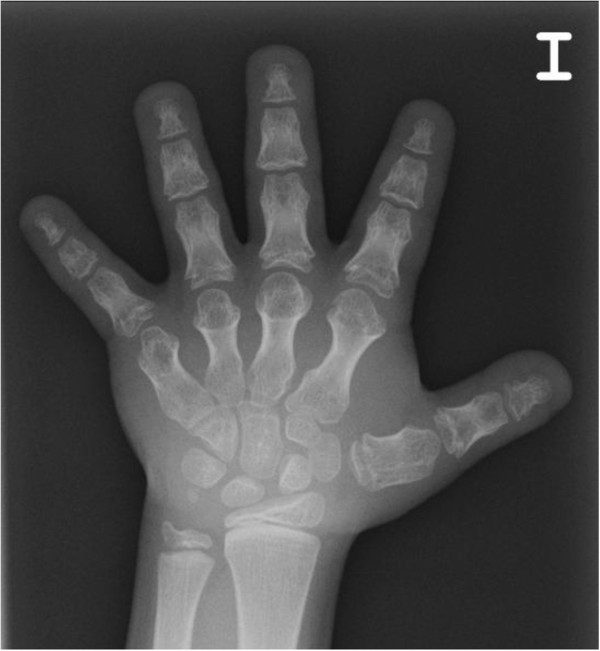
**Hand of a patient with acrodysostosis and multihormonal resistance (OMIM#101800).** Severe and generalized brachydactyly, through very short and broad tubular bones, including ulna can be observed. Metacarpals II-V are proximally pointed and cone-shaped proximal phalangeal epiphyses are prematurely fused. The general appearance of the hand is bulky and stocky (courtesy of Prof. Dr. Jesús Argente).

Acrodysostosis is a rare skeletal dysplasia characterized by severe generalized brachydactyly of hands (Figure 3) and feet with a relatively long first thumb, dysostosis, short stature and facial abnormalities: typically a round face with maxillary and nasal hypoplasia; but sometimes also an increased mandibular angle, mandibular prognathism, epicanthal folds and hypertelorism (see Figure Three Pt.7, 8, 11 at Linglart et al. [44]). Advanced skeletal maturation, spinal stenosis, obesity, mental deficiency, notably delayed speech and impaired hearing are also frequently observed (Additional file 1: Table S4) [44-51]. Although Maroteaux & Malamut defined this pathology as a new syndrome distinct from PHP/AHO in 1968 [47], these two entities have been confused many times because they share many features [44]. One of these is multihormonal resistance to PTH or TSH [52], though it is not always present as there are several forms of acrodysostosis and the genes involved are different (see below) [44]. In the case of acrodysostosis with multihormonal resistance (ACRDYS1, OMIM#101800), the gene responsible is cAMP-dependent protein kinase A (*PRKAR1A*), which has been localized to 17q24.2 and is involved in the signalling pathway downstream of *GNAS*. This is the reason for resistance to PTH and other hormones, as in PHP [44,48].

**Figure 4:**
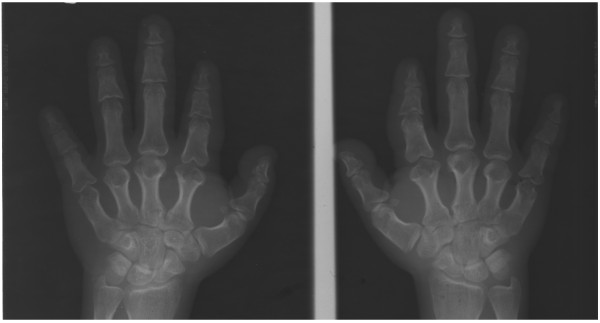
**Hands of a patient with Bilginturan BD or HTNB (OMIM%112410).** Metacarpals are short (specially II-V) with globular ends, irregular articular surfaces and cup deformity in the epiphysis of the proximal and medial phalanges. All the phalanges of the hands are also shortened, but the proximal and middle phalanges of the III and IV digits are relatively normal (courtesy of Dr. Sylvia Bähring and Dr. Okan Toka, unpublished case).

The characteristic feature in this syndrome is severe BDE, characterized by shortening of metacarpals/metatarsals and phalanges II-V, phalangeal epiphyses being cone-shaped and prematurely fused, and affected bones appearing bulky and stocky (Figure 4) [44,45,48], while thumbs and halluces tend to be less affected [44,52,53]. In addition, a decreased interpedicular distance has been reported in 75% of patients. These severe brachydactyly and vertebral abnormalities have not been noted in PHP patients, so they could help us to differentiate between these two syndromes [45,52]. Finally, another type of feature that could aid the differential diagnosis is subcutaneous ossification, as this is not seen in acrodysostosis [45,52].

### BDE without multihormonal resistance

#### Short stature

##### Bilginturan BD or hypertension with brachydactyly syndrome (HTNB, OMIM%112410)

Bilginturan *et al.* were the first to describe this syndrome in a large non-consanguineous Turkish family [54]. Subsequently, other authors have also studied this family and its new generations [55-66]. Affected members of the family had severe hypertension (30 mm Hg difference between affected and unaffected family members) from childhood [54] and a stroke by the age of 50 years if untreated [56]. Almost all the affected children have systolic blood pressure values over the 99th percentile [66]. However, the renin-angiotensin-aldosterone system (supine and upright) and circulating catecholamines respond normally, patients were not salt sensitive, their renal function was normal, they had no retinopathy or cardiac enlargement and the increase in radial wall thickness was minimal [55,56,59]. Affected individuals also presented BDE, were on average 10 cm shorter and had lower mean birth weight than their unaffected relatives, as well as having a stocky build and rather round face (see Figures two a & three a at Bilginturan et al. [54]) (Additional file 1: Table S5) [54,56,66].

Brachydactyly among the affected Turkish family members was heterogeneous. In most cases, shortening of more than one metacarpal and metatarsal was present [54], digits IV and V being most frequently affected but, in some cases, shortening has been seen in all the metacarpals and metatarsals in this and other families [56,57,66,67]. Further, the shortening is not always symmetrical. All the phalanges of the hand were shortened, but the proximal and middle phalanges of the III and IV digits were relatively normal. Phalanges revealed cone-shaped epiphyses at proximal ends. All the phalanges of the toes were shortened and some patients presented symmetrical fusion between the middle and distal phalanges of toes IV and V, or only V (Figure 4). Additionally, shortening of carpal bones in the axial direction was also observed [54]. Bähring *et al.* compared the brachydactyly pattern profile of a 5.5-year-old Japanese patient, who carried a *de novo* (12) (p11.21p12.2) chromosomal deletion [68], with a 6-year-old boy from the Turkish family [57]. Both boys presented almost identical brachydactylies; type 16 cone-shaped epiphyses, particularly in the proximal interpahalangeal joints of digits II and V, brachyphalangy of digits II-IV and brachymetacarpalia of digits IV and V. The Japanese patient presented brachymesophalangy and cone-shaped epiphyses in digits II-V, and the Turkish boy only in digits II and V [57]. Intellectual disability, subcutaneous calcifications, obesity or differences in body mass index have not been observed [55,57,66], except in an isolated case reported by Derbent *et al.*, where the patient presented obesity and a defect in the left renal artery [69]. It is important to highlight that brachydactyly is not appreciable until around 6 years of age and it aggravates significantly during early puberty as a result of an impaired growth spurt at the prepubertal stage. Given this, it is important to pay attention to the first signs of brachydactyly when an elevated blood pressure is detected in children (systolic blood pressure values >99th percentile) [66].

Another clinical feature in the Turkish family was the presence of aberrant posterior inferior cerebellar artery (PICA) loops only in affected members. Naraghi *et al.*[70] reported 100% loop cosegregation with hypertension and brachydactyly in a group of 27 members of the Turkish family (15 affected and 12 unaffected). The prevalence of the loops was not influenced by age or sex. Further, a brief report described a case of a boy with the same syndrome not only with the PICA abnormality but also abnormal renal arteries [71]. Additional studies found that affected individuals showed less active sympathetic nerve traffic to muscle than unaffected family members and also could not buffer blood pressure-elevating effects of phenylephrine [60,65]. These findings confirmed that baroreflex blood pressure buffering was impaired in affected individuals [60,66]. The arterial baroreflex is a critical cardiovascular reflex that provides continuous buffering of acute fluctuations in arterial blood pressure due, for example, to changes in posture, exercise, and emotion [72]. PICA loops can produce neurovascular compression of the ventrolateral medulla at the root entry zone of cranial nerves IX and X [63,70], which are very important in the baroreflex mechanism. Arterial baroreceptors are mechanoreceptors: those located in the carotid sinuses are innervated by cranial nerve IX and those in the aortic arch by cranial nerve X. When arterial pressure increases, these nerves send an input to the nucleus of the solitary tract and barosensitive neurons from here initiate a sympatho-inhibitory pathway to reduce arterial blood pressure [72].

The molecular basis for this syndrome is unknown, but several researchers have found evidence of linkage to chromosome 12p, specifically to 12p11.21-12p.12.2 [55,56,73,74]. Surprisingly, in a genome-wide linkage analysis, chromosome 12p was also implicated in Chinese patients with primary hypertension (not brachydactyly) [75]. Bahring *et al.* identified a complex rearrangement involving a deletion, reinsertion and inversion of the 12p12.2-p12.1 region [64]. This region is, however, relatively gene poor, only further characterized expressed sequence tags (EST) being identified in the vicinity [64]. These authors also proposed *Kir6.1*, *SUR2*, *LSOX5* and *PDE3A*, which are within the studied segment, as candidate genes. Although, no alterations were found in these genes, a higher mRNA expression of *PDE3A* was detected in older affected family members (while the younger subjects affected showed expression values in the same range as those unaffected). On this basis, they suggested that the higher vascular expression of *PDE3A* was probably not the cause of the hypertension, but rather the result of an increase in blood pressure with age [64]. Slightly different rearrangements in this segment were found later in other families, apart from the Turkish one [65]. Finally, in this inverted sequence, a non-protein coding exon was found only to be expressed in unaffected family members; the rearrangement could be the cause for this lack of expression. This transcript was predicted to be an miRNA expressed in primary fibroblasts [65]. The fibroblasts of affected family members showed faster cell growth, but the function of this miRNA remains to be clarified [65].

More severe cases caused by deletions in the 12p11.21-12p.12.2 region were reported by Lu *et al.*[74] and Nagai *et al.*[68]. One patient, with a deletion spanning 71 annotated genes (PDE3A→BICD1), had moderate mental retardation, short stature, borderline high blood pressure (120/80 mmHg) and characteristic brachydactyly [74]. Another patient presented a more severe phenotype, mild mental retardation, short stature, high blood pressure, brachydactyly and cone-shaped epiphyses of the hands, hypoplastic hair and skin, oligodontia, a small thoracic cage and a hypoplastic pelvis [68]. The difference in the severity between the patients might be attributable to the extent of the deletion [74].

##### BDE with short stature, *PTHLH* type (OMIM#613382)

Maass *et al.* were the first to implicate the pthyroid hormone-like hormone gene (*PTHLH*), localized to 12p11.22, in BDE, after the identification of a translocation, t(8;12) (q13;p11.2) in a family with autosomal-dominant BDE and other malformations, including short extremities, dysmorphic facies, macrocephaly, prominent forehead and a depressed nasal root [76]. Despite the *PTHLH* gene not being disrupted by the translocation, it produced a downregulation of the gene and consequently of its downstream targets, *ADAMTS-7* and *ADAMTS-12*[76]. These genes are highly expressed in chondrogenically differentiated fibroblasts, so they seem to play an important role in chondrogenesis [77,78]. In a more recent publication, Maass *et al.* proposed that the downregulation of *PTHLH,* and its targets, was due to the disruption of the interaction with a regulatory element localized 24.43 Mb downstream of *PTHLH, CISTR-ACT*[79]. This element is a *cis*-regulatory element of *PTHLH* and also a *trans*-regulatory element of *SOX9* (17q) [76,80]*,* which is another essential gene in chondrogenesis whose haploinsufficiency causes skeletal malformations [81]. This regulation occurs by an lncRNA (*DA125942*), derived from the *CISTR-ACT* locus, that coordinates the expression of *SOX9* and *PTHLH* generating a loop in the chromatin [79,80]. The translocations t(8;12) (q13;p11.2) and t(4;12) (q13.2-13.3;p11.2), later identified in another family, prevent the formation of such a loop and result in overexpression of the lncRNA and misexpression of *PTHLH* and *SOX9*[79,80].

In an independent study, a deletion and three point mutations in the coding sequence of *PTHLH* were found to be responsible for BDE with short stature [82]. Further, in an isolated case of symmetrical enchondromatosis caused by a *de novo* duplication of 12p11.23 to 12p11.22 including *PTHLH*, the patient showed BDE with cone-shaped phalangeal epiphyses [83].

**Figure 5:**
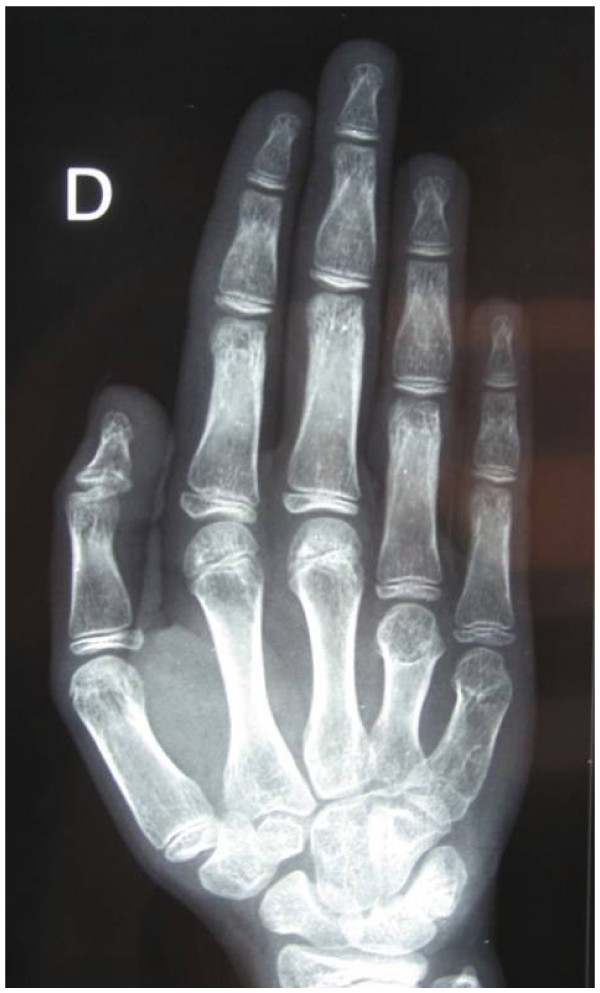
**Right hand in a non adult individual with heterozygous mutation in *PTHLH.*** Severe shortening of IV and V metacarpals is clearly observed (courtesy of Dr. Cécile Teinturier-Thomas, personal collection; Dr. Caroline Silve, molecular diagnosis, unpublished case).

As in the previous group, the pattern of brachydactyly varies considerably. Several authors have reported shortening of metacarpals III, IV and V in most cases, while proximal, middle and distal phalange involvement was variable and shortening and cone-shaped epiphyses on middle and distal phalanges II and V were frequently mentioned (Figure 5) [76,82,83]. In two cases (one patient with a deletion involving another 6 genes and the second a carrier of a small duplication), metacarpals I and II were also shortened [82,83], and in the case of translocation t(8;12) mentioned above, shortening of metacarpals I , III, IV and V was observed (Maass *et al.*; IV:2 patient) [76].

Apart from BDE, other features were seen in these patients (Additional file 1: Table S6): most but not all had short stature [76,82], tooth problems were reported by Klopocki *et al.* in two out of five families [82], and those affected with translocation t(8;12) had dysmorphic facies with macrocephaly [76]. Neither hypertension nor mental retardation was mentioned, but learning difficulties were reported in one family [82].

#### AHO-like

##### Brachydactyly mental retardation syndrome (BDMR, OMIM#600430)

Brachydactyly mental retardation syndrome (BDMR), also referred to as Albright hereditary osteodystrophy-like syndrome, is a rare pathology characterized by variable features: short stature, obesity, developmental delay, behavioural disorders, autism spectrum disorder and craniofacial and skeletal abnormalities, like BDE (see Figure one at Fernandez-Rebollo et al. [84]). Major malformations have also been reported, including cardiac, tracheal, gastrointestinal, genitourinary, CNS and skeletal abnormalities (Additional file 1: Table S7) [85,86].

This syndrome is caused by deletions in 2q37. Recently, the minimum critical region responsible for this syndrome has been mapped to the 200 kb which involve the histone deacetylase 4 gene (*HDAC4*), localized exactly to 2q37.3 [87]. In addition, point mutations in *HDAC4* have been found in two patients with BDMR [88]. On the other hand, a patient with a deletion in 2q37.3, but not including *HDAC4*, exhibited a similar phenotype but without BDE, cardiac or other major malformations [88]. Given this, it was proposed that *HDAC4* haploinsufficiency could be causative of BDE in this syndrome [88]. A recent report, of a family case with a microdeletion involving *HDAC4* and other two genes, puts this statement in doubt, because none of the three affected patients showed BDE [87]. So far, we know that BDE is a variable feature and only reported in about a half of patients with 2q37 microdeletions [87].

Histone deacetylase 4 is involved in bone, muscle, neurological and cardiac development [89]. Felder *et al.* demonstrated that *FARP2*, *HDLBP* and *PASK* genes were downregulated in a patient with a 2q37.3 terminal deletion compared to levels in his healthy family members and controls [90]. These are good candidate genes because they are involved in neuronal and/or skeletal development [90], but point mutations in these genes which cause BDMR or other similar syndrome have not yet been reported in the bibliography, except for the case of a patient with autism associated with a *de novo MECP2* duplication who also had a second *de novo* duplication of 2q37.3 material involving *PASK* and *HDLBP*. However, this patient did not have an AHO-like phenotype [91].

BDE is only penetrant in 50-60% of cases and the pattern profile is variable [88,92]. Frequently, metacarpals III and IV and metatarsal IV are shortened, but other metacarpals (II-V) have also been found to be affected, generally sparing metacarpal/metatarsal I [86,88,90,93-98]. Further, individuals with similar deletions or family members with the same deletion may present different phenotypes; this could be a result of incomplete penetrance of the haploinsufficiency genes, epigenetics or regulation by other genes [92].

It is worth mentioning that there is a very similar syndrome, but without BDE, known as Smith-Magenis syndrome (SMS; OMIM#182290) which is caused by deletions in 17p11.2 or mutations in the *RAI1* gene [88]. In addition*, RAI1* expression is reduced in BDMR cases [88]. Hence, when individuals are suspected of having the BDMR phenotype, but no 2q37 deletions or *HDAC4* mutations are detected, deletions at 17p11.2 and the *RAI1* gene should be analysed.

##### Acrodysostosis without multihormonal resistance (ACRDYS2, OMIM:#614613)

In acrodysostosis without hormonal resistance, the affected gene is the phosphodiesterase 4D, cAMP-specific (*PDE4D*) gene, localized to 5q11.2 [44,46,49,99]. Clinical features (Additional file 1: Table S4) are similar to ACRDYS1 apart from PTH resistance not generally being present [44,99], though an exception was reported by Michot *et al.*[46]. Other features it has been suggested may differ in the two types are: (i) facial dysostosis, which is more evident in ACRDYS2 than in ACRDYS1; and (ii) intellectual disability, which is common in ACRDYS2, while ACRDYS1 has only been associated with some behavioural disorders and/or learning difficulties [44,46]. On the other hand, in the study of Lee *et al.* all patients (three with *PDE4D* and two with *PRKAR1A* mutations) had midface hypoplasia and there were no differences in developmental disability according to the gene affected (among those with *PDE4D* mutations, one was not affected, a second was mildly affected and a third was significantly affected; whereas both patients with *PRKAR1A* mutations had mild developmental disabilities) [49]. Lynch *et al.* described eight patients; all of them had some degree of learning difficulties, developmental delay and intellectual disability [99]. These authors suggested that in patients with acrodysostosis associated with normal stature (only 5/15 presented short stature [46,49,99]), progressive obesity (frequently developing after 6 years of age), and mild intellectual disability but no PTH resistance, *PDE4D* rather than *PRKAR1A* mutations should be suspected. BDE, stenosis of the lumbar spine, nasal hypoplasia, a flat nasal bridge and short stature are features shared by both subtypes of acrodysostosis (see Figure three Pat. 15, 16 at Linglart et al. [44]). In addition, some cases of hypogonadism have been found with defects in either gene [44,49,99].

Thus, it is difficult to distinguish acrodysostosis cases with *PDE4D* mutations from those with *PRKAR1A* mutations on the basis of clinical observation only [49]. Therefore, biochemical studies of calcium and phosphorus metabolism are essential.

##### Tricho-rhino-phalangeal syndrome (TRPS)

This syndrome is classified into three types: TRPS I (OMIM#190350), TRPS II (OMIM#150230) and TRPS III (OMIM#190351). Mutations in the trichorhinophalangeal syndrome I gene (*TRPS1*)*,* localized to 8q23.3, have been found in 88% of cases of TRSP I and TRSP III [100]. Lüdecke *et al.* described TRPS III as an extreme of the clinical spectrum of TRSP I [100]. On the other hand, TRPS II is a contiguous gene syndrome involving *TRPS1* and *EXT1* genes; patients develop cartilaginous exostoses in addition to the features seen in TRPS I and III [100,101].

The striking features shared by the three types are sparse, slowly growing scalp hair, laterally sparse eyebrows, a bulbous tip of the nose (pear-shaped nose), long flat philtrum, thin upper vermillion border and protruding ears (see Figure three Momeni et al. [102]) (Additional file 1: Table S8) [100]. In radiography, the main findings are: short stature, BDE, mild metaphyseal convexity during the first year of life and premature fusion of the growth plates of the tubular bonds [100]. Additionally, hip malformations (coxa plana, coxa magna or coxa vara) have been reported in more than 70% of cases and degenerative arthrosis appears in older individuals [100]. Patients show a retarded skeletal age until puberty, and then an accelerated skeletal age, while the mean length at birth is normal [100]. That is, the growth retardation is a progressive process that can be appreciated in metacarpophalangeal pattern profile (MCPP) analysis comparing children and adults, and comparisons of their relative height [100]. Growth hormone deficiency has been reported in some cases [103-105]. Intellectual disability has also been reported in the three types, but most commonly in TRPS II [100,101,106,107].

**Figure 6:**
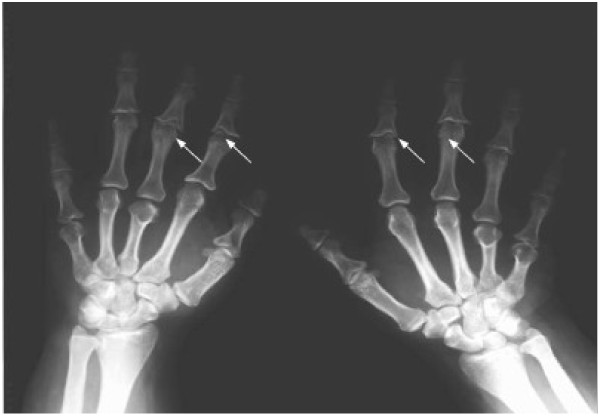
**Hands of a patient with tricho-rhino-phalangeal syndrome I (OMIM#190350).** The patient presents asymmetrical brachydactyly; metacarpals III and V are shortened on the left hand and IV and V on the right. Further, middle phalanges are shortened and cone-shaped epiphyses are shown with the typical outcarving and deformation (arrows) (courtesy of Dr. Sharona Azriel, unpublished case).

Regarding brachydactyly, shortening of the metacarpals has been reported in about half of patients and cone-shaped epiphyses of middle phalanges, type 12/12A (only appreciable after infancy in early childhood, before epiphyses fuse), in almost all patients, in most cases in mesophalanges II and III. Outcarving and deformation of the cones is another striking feature and this is more easily appreciated after epiphysis fusion (Figure 6) [108,109]. In addition, ivory cones were reported by Giedion (again, a feature that is only appreciable after infancy in early childhood, before epiphyses fuse). Hypoplasia of the thumb, as well as shortening of metacarpals II-V and of middle phalanges II and V is frequently seen, although shortening of all middle phalanges has been also reported [101,106,108,110], as have small feet and a short hallux [110]. Poznanski *et al.* conducted MCPP analysis comparing PHP and PPHP with TRPS and found no similarity between pattern profiles in the two syndromes [20]. These data were published before the association of the syndrome with the *TRPS1* gene. More recently, Lüdecke *et al.* used MCPP analysis to compare various groups of TRPS patients; first of all, they concluded that like height differences, the brachydactyly was more marked in adults than in children. On the other hand, patients with missense mutations (TRPS III) had more severe brachydactyly than the patients with the nonsense ones (TRPS I). Given this, they classified the brachydactyly pattern with respect to the mean profiles for TRSP I and TRSP III, but these mean values were only intended for classification not to be used as a tool for diagnosis [100]. Additionally, patients with missense mutations were shorter than the nonsense carriers, but patients with gene contiguous syndrome (TRPS II) were the smallest [100]. Finally, the brachydactyly pattern is variable, as reflected in a lack of correlation between MCPPs in patients with the same recurrent mutation as well as the presence of asymmetric brachydactyly in some patients [100].

The severity of the TRPS phenotype might also depend on the size of the deletion in 8q and whether there is mosaicism for the deletion, which has been reported in four patients, two with TRPS I and two with TRPS II [101].

##### Turner syndrome (TS)

Turner syndrome is a chromosomal disorder with a complete or partial monosomy of X chromosome. The genetic background is variable, the most frequent karyotypes are: 45,X (~50%), karyotypes with an isochromosome of X (i(Xq) or i(Xp)), the mosaic 45,X/46XX (%30), and karyotypes containing an entire or parts of Y chromosome [111,112].

The main features are short stature (90-100%), gonadal dysgenesis, pubertal delay, primary amenorrhea (85%), estrogens insufficiency, cardiac anomalies and/or other congenital malformations like brachydactyly E (see Figure one at Bondy [113]) [111,112,114-116]. Other frequent features are: lymphedema, congenital malformations of the urinary system (30-40%), abnormalities of the external ocular appendages, (epicanthal folds, ptosis, hypertelorism, upward slanting palpebral fissures), strabismus and hypermetropia (25–35%), hearing problems and ear malformations, dysmorphic craniofacial features, abnormalities in tooth development and morphology (early eruption), melanocytic nevi, normal GH secretory pattern, altered liver enzymes¸ decreased bone mineral density, etc. [111,112,114-117] (Additional file 1: Table S9).

**Figure 7:**
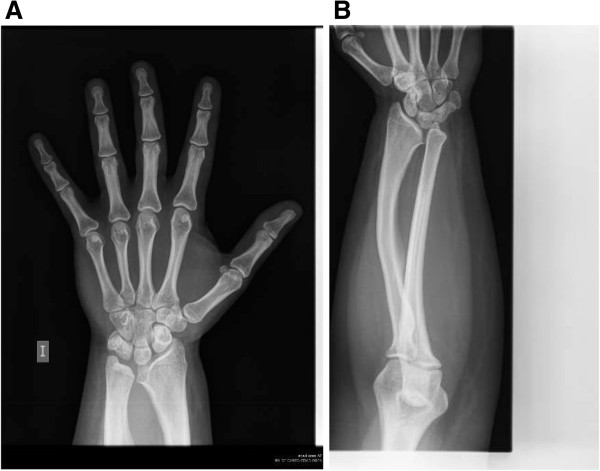
**Hand (A) and radius (B) of a patient with Turner syndrome.** It can be observed the ulnar and palmar slant of the radial articular surfaces, with a triangular appearance of the distal radial epiphysis and slight shortening of the IV metacarpal; the radius is short and bowed.

Regarding brachydactyly E, the more typical brachydactyly of Turner syndrome is the shortened of the IV metacarpals, but it is variable [20,114,118]. Poznanski compared the brachydactyly associated with PPHP-PHP *versus* TS and observed that the shortening of the bones are less severe in TS than in PPHP-PHP; shortening of I distal (D1) phalanx’s is less frequent in TS (5% in contrast to the 75% of PPHP-PHP), so if a patient presents short fourth metacarpals and a normal D1, it is more probable that have Turner syndrome than PHP-PPHP. Finally, he mentions drum stick phalanges and thin bones in TS (Figure 7) [20].

Although TS is quite well known entity, there is often a delay in the diagnosis as over 20% of patients are diagnosed after 12 yr [115,119]. As other syndromes mentioned in this review, it is essential to be diagnosed during infancy for proper early treatment to avoid complications linked to TS. In fact there are some publications explaining the clinical guidelines to be followed to optimize care for young woman and adults with TS [111,112,114-117].

## Concluding remarks

The BDE pattern profile can vary considerably within a syndrome, even between unrelated patients with identical mutations and among affected family members. In addition, brachydactyly is sometimes not appreciable until 6 years of age, because it is a progressive feature and tends to become more evident during early puberty.

**Figure 8:**
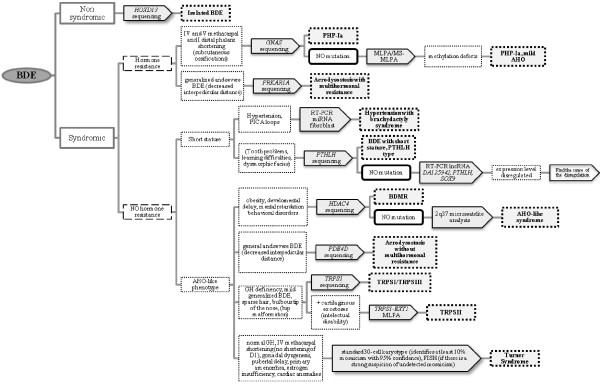
**Proposed clinical algorithm to suggest the most probable genetic diagnosis.** Features in brackets are not always present in the syndromes. *BDE* Brachydactyly type E, *PHP-Ia* Pseudohypopthyroidism type Ia, *MLPA* Multiplex ligation-dependent probe amplification, *MS-MLPA* Methylation specific-MLPA.

With the aim of helping to identify the most probable genetic diagnosis to inform the clinical management of the aforementioned syndromes with BDE, we have established a diagnostic algorithm including specific differential features (Figure 8). In a multidisciplinary approach for patients with BDE, there are some very subtle but helpful clues that allow us to distinguish between some of the syndromes. (i) BDE observed in individuals with acrodysostosis can be differentiated fairly clearly because of its severity: all the bones in the hand are affected and are characteristically stocky in appearance [44,45,48]. (ii) TRPS can also be easily recognised because the MCPP is significantly different and there are also other characteristic clinical features: sparse, slowly growing scalp hair and a bulb-shaped nose are very suggestive of this syndrome [20].

Poznanski et al. also carried out MCPP analysis comparing isolated BDE and PHP/PPHP, but at the time these two syndromes had not been genetically well differentiated and hence the conclusions may not be reliable [20]. Indeed, patients with different pathologies could be confused if attention were to be focused on radiographic findings alone; hence, biochemical analyses should be included.

The other syndromes discussed have not yet been compared by MCPP analysis, but it seems that the pattern of brachydactyly does not differ appreciably between them. In such cases, when the BDE pattern is not sufficiently informative, there are other features which can help in the differentiation of each syndrome (Additional file 1: Table S1). These features are listed in the supplementary material (Additional file 1: Table S2–S9).

## Competing interests

The authors declare that they have no competing interests.

## Authors’ contributions

AP and GPN designed the review. IG and EB participated in the molecular description of the syndromes. MGB and BG participated in the clinical description of the syndromes. MGB and AMI participated in the description of the radiological findings. All authors read and approved the final manuscript.

## Supplementary Material

Additional file 1: Table S1 Summary of description of each syndrome. ACRDYS: acrodysostosis; BDE: brachydactyly type E; BDMR: Brachydactyly mental retardation syndrome; PHP: pseudohypopthyrodism; TRPS: Tricho-rhino-phalangeal syndrome. **Table S2.** Summary of the phenotype associated with isolated BDE HOXD13 type [9-12]. **Table S3.** Summary of the phenotype associated with PHP-Ia [27,29]. **Table S4.** Summary of the phenotype of acrodysostosis [46,52,99]. **Table S5.** Summary of the phenotype associated with Bilginturan BD or hypertension with brachydactyly syndrome [54-66]. **Table S6.** Summary of the phenotype associated with BDE with short stature, PTHLH type [76,82]. **Table S7.** Summary of the phenotype associated with Brachydactyly mental retardation syndrome [86,88,90,92]. **Table S8.** Summary of the phenotype associated with TRPS [100,108]. **Table S9.** Summary of the phenotype associated with Turner syndrome [111,112,114-117]. Click here for file
