# Supplementary material for: Brachydactyly E: isolated or as a feature of a syndrome
Source: Orphanet J Rare Dis. 2013 Sep 12;8:141. doi: 10.1186/1750-1172-8-141 (PMC3848564; doi:10.1186/1750-1172-8-141)
Supplement: Additional file 1: Table S1 — Summary of description of each syndrome. ACRDYS: acrodysostosis; BDE: brachydactyly type E; BDMR: Brachydactyly mental retardation syndrome; PHP: pseudohypopthyrodism; TRPS: Tricho-rhino-phalangeal syndrome. Table S2. Summary of the phenotype associated with isolated BDE HOXD13 type [9-12]. Table S3. Summary of the phenotype associated with PHP-Ia [27,29]. Table S4. Summary of the phenotype of acrodysostosis [46,52,99]. Table S5. Summary of the phenotype associated with Bilginturan BD or hypertension with brachydactyly syndrome [54-66]. Table S6. Summary of the phenotype associated with BDE with short stature, PTHLH type [76,82]. Table S7. Summary of the phenotype associated with Brachydactyly mental retardation syndrome [86,88,90,92]. Table S8. Summary of the phenotype associated with TRPS [100,108]. Table S9. Summary of the phenotype associated with Turner syndrome [111,112,114-117]. [file 1750-1172-8-141-S1.doc]

Supplementary Table 1: Summary of description of each syndrome. ACRDYS: acrodysostosis; BDE: brachydactyly type E; BDMR: Brachydactyly mental retardation syndrome; PHP: pseudohypoparathyrodism; TRPS: Tricho-rhino-phalangeal syndrome

| **Syndrome** | omim | **Gene** | **Localization** | **Resistance** | **Brachydactyly pattern** | **Other features** |
| --- | --- | --- | --- | --- | --- | --- |
| **ACRDYS1** | OMIM#101800 | *PRKAR1A* | 17q24.2 | PTH, TSH | Metacarpal and phalanges II-V of hands and foot | Short stature, obesity, nasal hypoplasia, flat nasal bridge, advanced skeletal maturation, spinal stenosis, hearing loss and mental retardation, hypogonadism |
| **ACRDYS2** | OMIM:#614613 | *PDE4D* | 5q11.2 | NO |  |  |
| **BDE with short stature, PTHLH type** | OMIM#613382 | *PTHLH* and *DA125942* lncRNA | 12p11.22 | NO | Metacarpals: III, IV, V  Phalanges: middle and distal involvement is variable. Middle and distal phalanges shortening of digits II and V and cone-shaped epiphyses is common | Short stature¸ tooth problems, (dysmophic facies, learning difficulties) |
| **BDMR** | OMIM#600430 | 2q37/*HDAC4* | 2q37.3 | NO | Metacarpals: III, IV, sometimes II-V Metatarsal: IV  sparing metacarpal/metatarsal I | Short stature, obesity, rounded face, mild-moderate mental retardation |
| **Hypertension with BDE** | OMIM%112410 | miRNA (12p12.2-p12.1) | 12p11.21-12p.12.2 | NO | Metacarpals: IV, V Phalanges: all but the proximal and middle phalanges of the III and IV digits relatively normal.  Type 16 cone-shaped epiphyses, particularly in the proximal interpahalangeal joints of the II and V digits. | Hypertension, 10 cm on average shorter than unaffected family members, lower birth weight mean, stocky build, rather round face |
| **Isolated BDE** | OMIM#113300 | *HOXD13* | [2q31.1](http://omim.org/geneMap/2/567?start=-3&limit=10&highlight=567) | NO | Metacarpals: III or/and IV; Metatarsals: IV; sometimes I, III or V. Broadening of the hallux. Little finger distal phalanx hypoplasia/aplasia, finger IV: lateral phalangeal duplication and/or clinodactyly, and fingers III/IV syndactyly | Generally, normal stature, normal psychomotor development |
| **PHP** | OMIM#103580 | *GNAS* | 20q13.2-20q13.3 | PTH, TSH… | Metacarpals: III, IV, V  Phalanges: I distal | Short stature, obesity, rounded face, subcutaneous calcifications, mental retardation |
| **TRPS I/III** | OMIM#190350  OMIM#190351 | *TRPS1* | 8q23.3 | NO | Hypoplasia of the thumb  Metacarpals: II-V  Phalanges: II, V middle. Sometimes shortening of all middle phalanges  Type 12/12A cone-shaped epiphyses in middle phalanges, in most cases in mesophalanges II and III. | Short stature, sparse, slowly growing scalp hair, laterally sparse eyebrows, a bulbous tip of the nose, long flat philtrum, upper vermillion border and protruding ears, hip malformations, retarded skeletal age until puberty, and then an accelerated skeletal age |
| **TRPS II** | OMIM#150230 | *TRPS1*-*EXT1* |  | NO |  | TRPSI/III features + cartilaginous exostoses |
| **Turner Syndrome** |  | X0 |  | NO | Metacarpals: IV | Short stature, gonadal dygenesis, pubertal delay, primary amenorrhea, estrogens insufficiency, cardiac anomalies |

**Supplementary Table 2.** Summary of the phenotype associated with isolated BDE *HOXD13* type [9-12]

*Radiological findings*

BDE

Digit V, distal phalamgeal hypoplasia/aplasia

Lateral phalangeal duplication and/or clinodactyly of finger IV

III/IV finger syndactyly

IV/V toe syndactyly with partial or complete digit duplication

**Supplementary Table 3.** Summary of the phenotype associated with PHP-Ia [27,29].

*Clinical features*

Rounded face

Short stature

Central obesity

Variable degrees of mental retardation (adult 27% and the pediatric population 64%)

Resistance to hormones PTH, TSH, gonadotropins, and GHRH (variable severity)

Hypocalcaemia, hyperphosphataemia, and elevated circulating PTH

Hypogonadism, delayed or incomplete sexual maturation, slightly hypoestrogenic, and/or infertility (particularly females)

Amenorrhea or oligomenorrhoea

Prolactin deficiency

*Radiological findings*

BDE

Subcutaneous ossifications

**Supplementary Table 4**. Summary of the phenotype of acrodysostosis [46,52,99].

*Clinical features*

Prenatal onset of skeletal dysplasia

Short stature

Broad face and widely-spaced eyes

Maxillonasal hypoplasia, flattening of nasal ridge, small and upturned nostrils

Prominent mandible

Hypoplastic ear helices, prominent ears

Peripheral dysostosis

Mental retardation/delayed development (77-80%)

Recurrent otitis media and hearing loss (30–67%)

Epicanthic folds (39%)

Hypogonadism (cryptorquidism, irregular menses, small hypoplastic genitalia) (29%)

Dental abnormalities (delayed tooth eruption/hypodontia) (26%)

Renal abnormalities (3%)

Limitation of extension of elbows

Enthesopathy

Obesity

Scoliosis

Heterochromia of iris

Eczema, sleep apnoea, rhinitis

*Radiological findings*

Severe hypoplasia of the skull

Thickened calvarium

BDE affecting all metacarpals/metatarsals and phalanges of digits II-V

Cone-shaped epiphyses with early epiphyseal fusion in hands and feet

Hyperplasia of the metatarsal and phalanges of the hallux

Decreased interpedicular distance and widening in the cephalocaudal direction

Increased mandibular angle (68–81%)

**Supplementary Table 5.** Summary of the phenotype associated with Bilginturan BD or hypertension with brachydactyly syndrome [54-66]

*Clinical features*

Severe hypertension (30 mm Hg difference between affected and unaffected family members)

Stroke

Aberrant posterior inferior cerebellar artery (PICA) loops (100%)

Short stature

Lower mean birth weight

Stocky build

Rather round face

Renal arteries abnormalities (one case)

*Radiological findings*

BDE

Cone-shaped epiphyses

**Supplementary Table 6**. Summary of the phenotype associated with BDE with short stature, PTHLH type [76,82]

*Clinical features*

Short stature

Short extremities

Dysmorphic facies

Macrocephaly

Prominent forehead

Depressed nasal root

Tooth anomalies

*Radiological findings*

BDE

Cone-shaped epiphyses

**Supplementary Table 7.** Summary of the phenotype associated with Brachydactyly mental retardation syndrome [86,88,90,92]

*Clinical features*

Sparse hair (20%); sparse, arched eyebrows

Rounded face or full cheeks; flat mid-face; microcephaly (10%)

Deep set eyes, up-slanting palpebrae

Short nose, deficient, notched nares, low-set columella

Short philtrum, hypoplastic Cupid´s bow of upper lip

Pinna anomalies (minor)

Inverted nipples

Major malformations (30%): Cardiac (20%), tracheal, gastrointestinal (11%), genitourinary common (11%)

CNS, skeletal less common

AHO-like phenotype; obesity, short stature (23%)

Hypotonia; connective tissue phenotype, joint laxity, umbilical and inguinal hernias (29%)

Mild-moderate mental retardation, speech delay

Autism (24%), behaviour disorders, seizures (35%), epileptic (25%), episodes of depression, hyperactivity

Eczema, asthma, recurrent infections, Wilms tumour, cystic kidneys

*Radiological findings*

BDE in hands and feet (50-60%)

**Supplementary Table 8**. Summary of the phenotype associated with TRPS [100,108].

*Clinical features*

Sparse, slowly growing scalp hair

Medially thick, laterally thin and sparse arched eyebrows

Bulbous tip of the nose with a broad and prominent nasal root

Long flat philtrum

Upper vermillion border

Protruding ears

Seizures

Web-shaped neck

Mild metaphyseal convexity

Premature fusion of the growth plates of the tubular bonds

Hip malformations (70%); coxa plana, coxa magna or coxa vara

Osteonecrosis of the femoral head

Cardiopathies

Renal diseases

Intellectual disability

*Radiological findings*

Hypoplasia of the thumb, shortening of II-V metacarpals and middle phalanges

Nonsymmetric brachydactyly

Type 12/12A midphalangeal cones

Clinodactyly of digits III, IV

Finger nail abnormalities

Small feet and short hallux

Triangular distal ulnar epiphyses

Sella turcica bridge

Cartilaginous exostoses (TRPSIII)

**Supplementary Table 9.** Summary of the phenotype associated with Turner syndrome [111,112,114,114-117]

*Clinical features*

Short stature (>50%)

Normal GH secretory pattern

Prominent ears (>50%)

Retrognatia (>50%)

Narrow palate (>50%)

Cubitus valgus (25-50%)

Ptosis (25-50%)

Strabismus (25-50%)

Epicanthal folds (10-25%)

Hypertelorism

Upward slanting palpebral fissures

Abnormalities in tooth development and morphology

Early eruption of the secondary teeth

Scoliosis (10-25%)

Kyphosis (10-25%)

Pectus excavatum (10-25%)

Flat feet (10-25%)

Genu valgum (<10%)

Madelung deformity (<10%)

Patellar dislocation (<10%)

Growth failure (>50%)

Chronic otitis media (>50%)

Low BMD (>50%)

Fractures (>50%)

Feeding problems (25-50%)

Sensonneural hearing loss (25-50%)

Opstructive sleep apnea (10-25%)

Articulation problems (10-25%)

Hyperacusis (<10%)

Low posterior hairline (>50%)

Lymphedema (>50%)

Nail dysplasia (>50%)

Webbed neck (25-50%)

Single palmar crease (10-25%)

Inverted nipples (10-25%)

Infertility (>50%)

Gonadal failure (>50%)

Delayed puberty (>50%)

Learning disability (>50%)

Renal malformation (25-50%)

Hypertension (25-50%)

Multiple nevi (25-50%)

Hypothyroidism (10-25%)

Cardiac problems (10-25%)

Diabetes mellitus (10-25%)

Celiac disease (<10%)

Inflammatory bowel disease (<10%)

Von Wilebrand’s disease (<10%)

JRA (<10%)

Pilomatrixoma (<10%)

Liver enzymes (glutamyl transferase, alanine amino transferase, aspartate amino transferase, and alkaline phosphatise) commonly raised

*Radiological findings*

BDE (short IV methacarpals) (25-50%)
